# Identification and function characterization of NcAP2XII-4 in *Neospora caninum*

**DOI:** 10.1186/s13071-024-06477-1

**Published:** 2024-09-14

**Authors:** Huizhu Nan, Xin Lu, Chao Zhang, Xin Yang, Zhu Ying, Lei Ma

**Affiliations:** 1 Ministry of Education Key Laboratory of Molecular and Cellular Biology, College of Life Sciences, Hebei Normal University, Shijiazhuang, 050024 Hebei China; 2 Hebei Collaborative Innovation Center for Eco-Environment, Shijiazhuang, Hebei China; 3 Hebei Key Laboratory of Animal Physiology, Biochemistry and Molecular Biology, College of Life Sciences, Hebei Normal University, Shijiazhuang, Hebei China; 4 National Animal Protozoa Laboratory, College of Veterinary Medicine, China Agricultural University, Beijing, 100193 China

**Keywords:** Neospora caninum, NcAP2XII-4, DAP-Seq, Transcription factor

## Abstract

**Background:**

*Neospora caninum* is a protozoan parasite in the Apicomplexa controlled by complex signaling pathways. Transcriptional control, an important way to regulate gene expression, has been almost absent in the *N. caninum* life process. However, to date, research on the transcriptional regulation of the AP2 family factors in *N. caninum* has been extremely limited. A prior study demonstrated that removing rhoptry protein 5 (ROP5), a significant virulence factor, resulted in abnormal expression levels of predicted NcAP2XII-4 in *N. caninum*, suggesting that the factor may regulate the function of ROP5. This study aimed to identify NcAP2XII-4 and its function in transcriptional regulation.

**Methods:**

The NcAP2XII-4 gene was identified by analyzing the *N. caninum* genome. A polyclonal antibody against the protein was prepared and purified, and its expression and localization in the parasite were detected using western blot (WB) and immunofluorescence assay (IFA). The ΔNcAP2XII-4 strain was constructed from the Nc1 strain using CRISPR/Cas9 to study its effect on the growth and development of *N. caninum*, and DAP-Seq and electrophoretic mobility shift assay (EMSA) were used to verify the transcriptional regulatory functions of the gene.

**Results:**

Bioinformatic analysis showed that NcAP2XII-4 consists of 11,976 bp and encodes 3991 amino acids, with a predicted molecular mass of 410 kDa. The protein has two AP2 domains, 1207aa-1251aa and 3453aa-3500aa, and is predicted to be located in the nucleus. The results of PCR, WB, and IFA were in accordance with the bioinformatics analysis. ΔNcAP2XII-4 was successfully constructed, but the strain could not be released and ultimately succumbed within parasitophorous vacuoles (PVs). Plaque assays demonstrated that parasites lacking this gene could not form plaques. One motif was successfully identified using DAP-Seq technique. Two prokaryotic expression vectors containing the AP2 domain of NcAP2XII-4 were successfully constructed, and two prokaryotic expression proteins, AP2-D1 and AP2-D2, and ROP5 biotinylated probes were prepared. Using EMSA, NcAP2XII-4 was shown to regulate ROP5 transcription by binding to its promoter.

**Conclusions:**

NcAP2XII-4 is an essential gene in *N. caninum*. This study provides a foundation for further research on transcriptional regulation in *N. caninum* and identifies a new candidate factor for the development of vaccines against *N. caninum*.

**Graphical Abstract:**

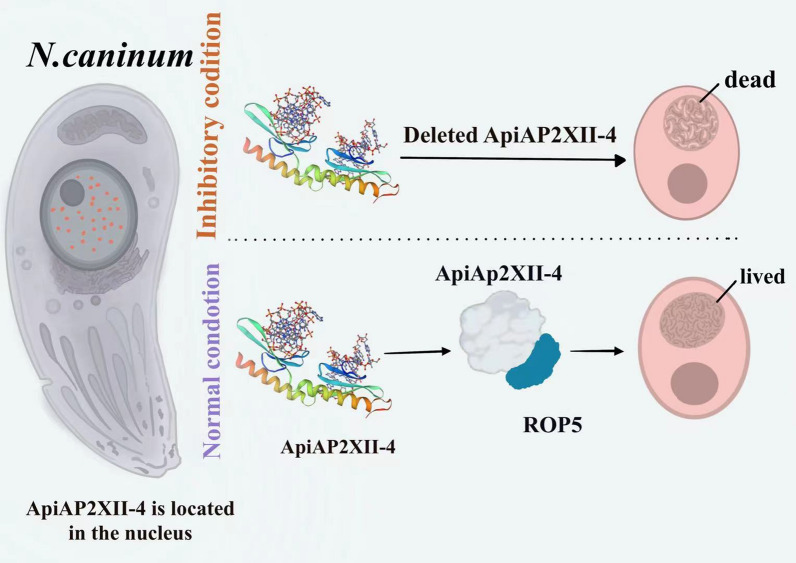

## Background

Neosporosis, caused by the protozoan parasite *Neospora caninum*, poses a significant threat to animal husbandry. This apicomplexan intracellular parasite primarily infects canids as its definitive host and a variety of other animals as intermediate hosts [1]. The disease manifests through symptoms, such as abortion, stillbirth, mummification, and neurological dysfunction [2]. Despite the severity of neosporosis, effective vaccines for *N. caninum* remain elusive. Inactivated vaccines, recombinant protein vaccines, and live vaccines [3–5] have not been used in the field. To combat this disease, further research is needed.

AP2 transcription family factors are important for plant growth, reproduction and interactions with the environment. Balaji et al. [6] found a domain in Apicomplexan protozoa that was similar to the AP2 superfamily protein in plants. The corresponding genes were subsequently named Apicomplexan AP2 (ApiAP2). Tracey et al. [7] comprehensively surveyed the DNA-binding specificities of 27 ApiAP2 protein families in *Plasmodium falciparum* and demonstrated that ApiAP2 proteins modulate key regulatory decisions throughout parasite development. For example, AP2-O is involved in biological processes including morphogenesis, locomotion, midgut penetration, protection against mosquito immunity, and preparation for subsequent oocyst development [8]. AP2-I is a key regulator of red blood cell invasion [9], while AP2-FG regulates female-specific gene expression [10]. AP2-G5 is essential to gametocyte maturation [11], and AP2-G triggers the initiation of differentiation and early development of gametocytes in the late trophozoite stage [12]. Moreover, AP2-O, AP2-SP, AP2-HC and AP2-G2 were studied in *Plasmodium* [13, 14]. Timothy et al. [15] described a novel set of antimalarial compounds that interact with the AP2 DNA-binding domains. ApiAP2 factors have been found in other apicomplexans and have been verified to bind to DNA containing specific motifs, acting as transcription factors. In *Toxoplasma*, 67 ApiAP2 proteins have been identified [16, 17]. Many studies have been conducted on the function of many ApiAP2 transcription factors in *Toxoplasma gondii* [18–20]. Many factors are related to parasite virulence; cell division cycle; development of tachyzoites, bradyzoites, and cysts; and sexual stage regulation of the parasite [17, 19, 21–25]. Fifty-three ApiAP2 transcription factors have been predicted in *Eimeria tenella* [26], but their functions have not been studied in depth. Members of the ApiAP2 transcription factor family play important roles in regulating life cycle transitions and other aspects of parasitic biology. Understanding the intricate roles of ApiAP2 factors in apicomplexan parasites holds great promise for advancing our knowledge of parasitic biology and developing effective interventions.

Deletion of the key virulence factor, rhoptry protein 5 (ROP5), leads to abnormal expression levels of predicted NcAP2XII-4, NcAP2XI-5, and NcAP2VII-2 in *N. caninum*, suggesting that these factors are related to the regulation of the parasite life cycle [27]. The function of these factors, especially in NcAP2XII-4, has been rarely studied in *N. caninum*, even across all parasites. Therefore, this study attempted to identify and explore the function of *N. caninum* NcAP2XII-4. This will lay the foundation for further research on the transcriptional regulation of *N. caninum* by ApiAP2 proteins and provide new directions for the development of anti-*N. caninum* drugs.

## Methods

### Parasite strains and cell culture

The *N. caninum* Nc1 strain used in this study was a gift from Professor Qun Liu (College of Veterinary Medicine, China Agricultural University) and stored at the College of Life Sciences, Hebei Normal University. All parasites were maintained in confluent monolayers of human foreskin fibroblasts (HFFs). HFF cells were cultured in DMEM supplemented with 20% fetal bovine serum (FBS). *Neospora caninum* tachyzoites were inoculated into cells and cultured with DMEM supplemented with 2% FBS at 37 ºC with 5% CO_2_.

### Bioinformatic analysis of NcAP2XII-4

The protein sequence of NCLIV_063920 (NcAP2XII-4) was obtained from the ToxoDB (http://toxodb.org/toxo/) website. The physical and chemical properties of *N. caninum* NcAP2XII-4 were obtained using the online tool ExPASy-Protparam (https://web.expasy.org/protparam/), subcellular localization by Cello v2.5, transmembrane structure by TMHMM 2.0, signal peptide by SignalP-4.1 Server, the secondary structure by SOPMA, the tertiary structure by SWISS-MODEL, and phosphorylation site by Netphos 3.1 Server.

### Amplification of the *NcAP2XII-4* gene

Total RNA was extracted from 1 × 10^8^ tachyzoites of *N. caninum* Nc1 using TRIzol reagent and converted to cDNA using an EasyScript First-Strand cDNA Synthesis SuperMix kit (TransGen, Beijing, China), in accordance with the manufacturer’s instructions. The open reading frame was amplified by PCR using the primers NcAP2XII-4-F/R (F: 5′-ATGGAGCACTGGGACTTGGGCGAGGAC-3′/R: 5′-GTGGAGGTCCAGGGTTGCAGATGCG-3′). The product was cloned into the *pEASY*^®^-Blunt Cloning Vector (TransGen Biotech, Beijing, China), and the recombinant plasmid was identified by PCR and sequencing.

### Preparation of NcAP2XII-4 polypeptide antibody

One antigenic sequence containing 12 amino acids (C-PETSRAGSSGVA) was designed for NcAP2XII-4 polypeptides. The polypeptide was synthesized using the typical solid-phase synthesis and qualified by HPLC–MS to ensure 95% purity. The polypeptide was conjugated to keyhole limpet hemocyanin (KLH) at the N-terminus. New Zealand White rabbits were immunized with NcAP2XII-4-KLH conjugates to prepare anti-NcAP2XII-4 polypeptide antibodies. Two rabbits were each vaccinated with a dose of 500 µg polypeptide per session by a single subcutaneous injection at 2-week intervals. Serum was obtained after five immunizations and stored at −80 ºC after purification.

### Western blot (WB) and immunofluorescence assay (IFA)

For WB, 1 × 10^8^ tachyzoites were collected, and the nuclear protein was extracted using the *ProteinExt*^®^ Mammalian Nuclear and Cytoplasmic Protein Extraction Kit (TransGen Biotech, Beijing, China), according to the manufacturer’s instructions. The proteins were separated using an 8% SDS-PAGE and then transferred onto a polyvinylidene fluoride (PVDF) membrane. The specific primary antibody, anti-NcAP2XII-4, was added at a dilution of 1:100 and incubated overnight at 4 ºC, followed by incubation with HRP-labeled goat anti-rabbit IgG (TransGen Biotech, Beijing, China). The bands were visualized using a highly sensitive ECL luminescence reagent (Sangon Biotech, Shanghai, China). IFA was used to detect the NcAP2XII-4 subcellular localization in *N. caninum*, as previously reported [27]. The difference was the use of primary and secondary antibodies. The primary antibodies used were rabbit anti-NcAP2XII-4 (1:100) and mouse anti-NcACP (1:100). The secondary antibodies used were Cy3-labeled donkey anti-rabbit IgG (1:100, TransGen Biotech, Beijing, China) and FITC-labeled goat anti-mouse IgG (1:100, TransGen Biotech, Beijing, China).

### Phylogenetic analysis

Nucleotide sequences were subjected to correction before BLAST alignment (http://toxodb.org/toxo/ and https://blast.ncbi.nlm.nih.gov/Blast.cgi). All sequences downloaded from GenBank were aligned using MEGA 6.06 (https://www.megasoftware.net) and edited manually (available on request). Bayesian analysis was performed using MrBayes 3.0 (http://nbisweden.github.io/MrBayes/) with four MCMC strands, 100,000 generations, and an initial burn-in of 1000. Four categories of among-site rate variations were used, and trees were sampled every ten generations.

### Construction of the NcAP2XII-4 knockout *N. caninum* strain

The CRISPR/Cas9 system was used to generate NcAP2XII-4 deficiency (∆NcAP2XII-4 parasites) as previously described [28]. The EuPaGDT Library in ToxoDB (https://toxodb.org) was used to design the gRNA targeting sites of the plasmids pCRISPR-CAS9-AP2XII-4. The Cas9 upstream and downstream fragments containing gRNA sequences were amplified and ligated using a multi-fragment connection kit (TransGen Biotech, Beijing, China). To disrupt the NcAP2XII-4 locus, the chloramphenicol resistance gene (CAT) was inserted into the 3′ and 5′ flanks of the NcAP2XII-4 region and ligated into the plasmid backbone carrying ampicillin resistance. To construct homologous recombinant plasmids of NcAP2XII-4 (pAP2XII-4-CAT), the 3′ flanking and 5′ flanking sequences of the NcAP2XII-4 gene were amplified from the genomic DNA of Nc1 parasites. The pCRISPR-CAS9-AP2XII-4 and pAP2XII-4-CAT plasmids were co-transfected into Nc1 parasites. Thereafter, the parasites were cultured and screened using chloramphenicol. The resulting strain was named ΔNcAP2XII-4.

### Plaque assay

Experiments were performed as previously reported [27]. HFFs had been previously cultured in six-well plates. Subsequently, 300 Nc1 tachyzoites per well were inoculated into the cells. Concurrently, all ΔNcAP2XII-4 tachyzoites, which had been transfected with a knockout plasmid, were infected into the cells. The plate was incubated for 7 days in a 37 ºC incubator with 5% CO_2_, fixed with 4% paraformaldehyde for 20 min, and stained with 2% crystal violet staining (Sangon Biotech, Shanghai, China). The stained wells were visualized under a fluorescence microscope (Leica Microsystems, Germany). The plaque area measurement was performed as previously described [29].

### Screening of NcAP2XII-4 binding sites

DAP-seq was performed as previously described [30]. Tachyzoites (1 × 10^9^) were collected and purified, and then genomic DNA was extracted using a genomic DNA kit (TransGen Biotech, Beijing, China). A DNA library was constructed by fragmentation, recovery, end repair, and joining in turns. The pDAP-Halo-AP2XII-4 expression vector was constructed and subsequently expressed in a wheat germ expression system provided by Promega. Upon binding the NcAP2XII-4 protein to the prepared DNA library, high-throughput sequencing was performed using an Illumina HiSeq (Zoonbio, Jiangsu, China). The data obtained were aligned with the *N. caninum* genome using BWA-MEM. All reliable peaks were obtained in the two independent biological replicates by combining both the MACS call peak and IDR software. The peaks were annotated using HOMER software. The conserved motifs were analyzed using MEME software. The frequency distribution of reads near the TSS was analyzed using depTools2 software. Gene Ontology and Kyoto Encyclopedia of Genes and Genomes (KEGG) enrichment analyses were performed on genes associated with the peaks using the Gene Ontology Resource (https://geneontology.org/) and KEGG (http://www.genome.jp/).

### Electrophoretic mobility shift assay (EMSA)

To validate the binding of the NcAP2XII-4 to the ROP5 gene promoter, two sequences of the AP2 domain of NcAP2XII-4, AP2-D1, and AP2-D2 were amplified from *N. caninum* cDNA. The sequenced AP2-D1 and AP2-D2 sequences were subcloned separately into the pET30a expression vector containing His and Halo-tag to create the pET-Halo-AP2-D1 and pET-Halotag-AP2-D2 plasmids. The correct pET-Halo-AP2-D1 and pET-Halotag-AP2-D2 plasmids were transformed into chemically competent Transetta (DE3) cells (TransGen Biotech, Beijing, China) for protein production. Protein expression was then induced with 0.8 mM IPTG for 4 h at 37 °C. Following the protocol of the Ni–NTA resin (TransGen Biotech, Beijing, China), proteins were extracted and purified after the bacteria had been harvested. Subsequently, they were detected by SDS-PAGE. Biotin-labeled DNA probes measuring 28 bp in length (probe F:5′-GGCACTTCTTCAACTGACAACGTAATCA-3′, probe R:5′-TGATTACGTTGTCAGTTGAAGAAGTGCC-3′, probe F (cold): 5′- GGCACTTCTTCAACTGACAACGTAATCA-3′) were designed and synthesized for use in EMSA. EMSA reactions were performed according to the manufacturer’s instructions for the Lightshift Chemiluminescent EMSA Kit (Thermo Scientific) [31]. Finally, the signals were detected using a fluorescence/chemiluminescence imaging system (ChemiScope 6000Pro; CLiNX, Shanghai, China).

## Results

### Bioinformatics analysis of NcAP2XII-4

**Fig. 1:**
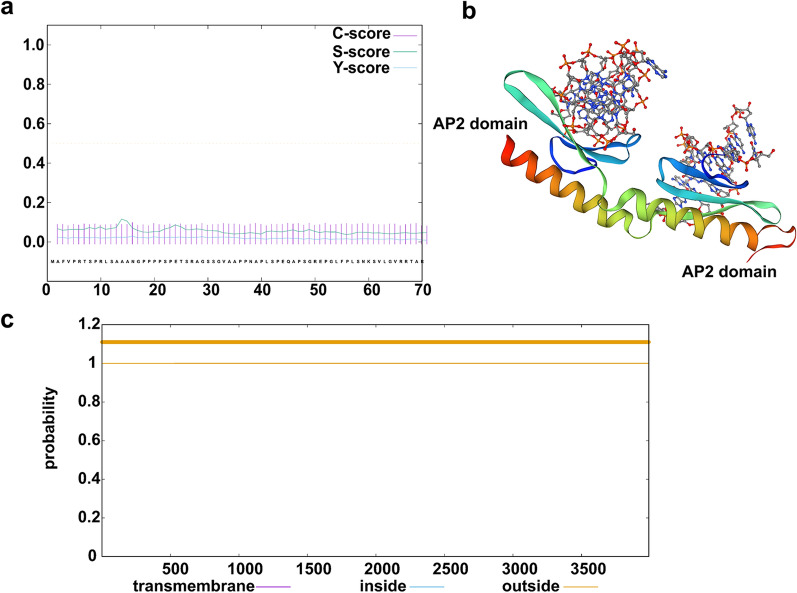
Bioinformatic analysis of *Neospora caninum* transcription factor NcAP2XII-4. **a** Prediction of the signal peptide structure of NcAP2XII-4. **b** Prediction of the domain structure of NcAP2XII-4. **c** Prediction of the transmembrane region of the NcAP2XII-4

Bioinformatic analysis showed that the full-length sequence of *N. caninum* NcAP2XII-4 was 12,259 bp, including one intron and two exons. The length of coding gene was 11,976 bp, which encoded 3991 amino acids. The molecular weight of the protein was approximately 410 kDa, with an instability coefficient of 55.69 and hydrophilicity of – 1.063. These characteristics indicate that it is a hydrophilic protein with an isoelectric point of 7.06. This protein did not contain any signal peptides (Fig. 1a) or transmembrane regions (Fig. 1c). The phosphorylation sites of a protein play important roles in predicting its potential function. The predicted analysis indicated that there were 171 phosphorylation sites, consisting of 106 serine sites, 64 threonine sites, and one tyrosine site. Subcellular localization analysis suggested that the protein was located in the nucleus with a reliable index of 3.731 and high credibility. Sequence alignment and modeling analysis showed that *N. caninum* NcAP2XII-4 contains two AP2 domains, 1207aa-1251aa and 3453aa-3500aa, comprising one alpha-helix and three beta-folded structures in each domain (Fig. 1b). These structures exhibit similarities to the classical model PF140633 AP2 domain. The two AP2 domains are linked through an alpha helix to bind with DNA and exert transcriptional regulatory function, suggesting that *N. caninum* NcAP2XII-4 could play a certain role in the transcriptional regulation in the life cycle of the parasite.

### Identification of *N. caninum* NcAP2XII-4

**Fig. 2:**
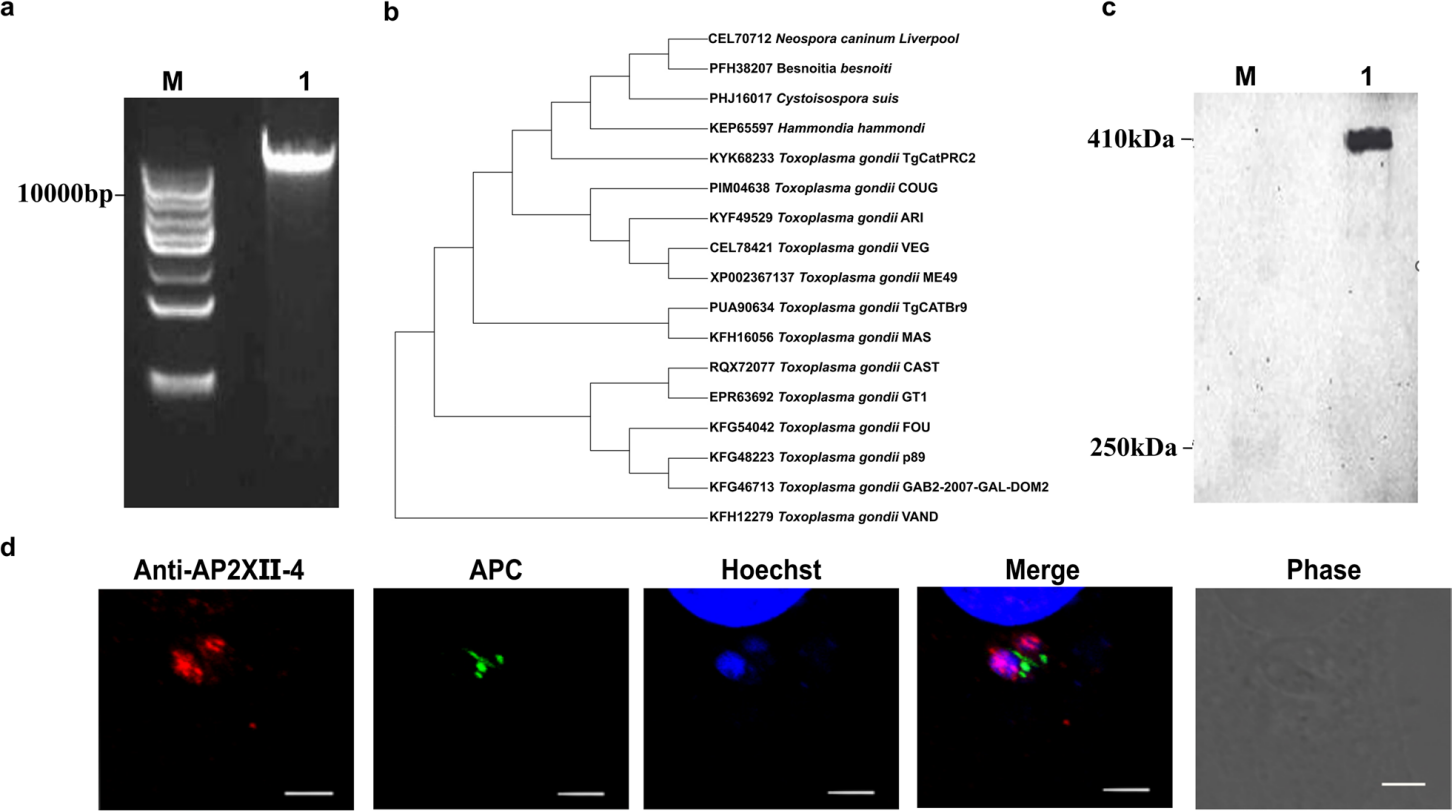
Identification of NcAP2XII-4. **a** Amplification of NcAP2XII-4 gene. **b** Phylogenetic analysis of NcAP2XII-4 gene evolution in the apicomplexan classes. **c** Western blot results of the NcAP2XII-4 antibody. **d** Immunofluorescence localization of NcAP2XII-4 protein in *Neospora caninum*. Scale bar, 5 µm

Three fragments were successfully amplified using *N. caninum* cDNA as a template. Additionally, a fragment of approximately 12,000 bp was amplified, and the fragment matched the predicted gene length of NcAP2XII-4 (Fig. 2a). DNA sequence assays showed that the full-length *N. caninum NcAP2XII-4* was successfully obtained, and the sequence exhibited 100% identity with the known sequence. According to the results of the phylogenetic analysis, *N. caninum* NcAP2XII-4 exhibits a greater amino acid sequence identity with *Besnoitia besnoitai*, *Cystoisospora suis*, and *Hammondia hammondi* (Fig. 2b). Western blot showed that the polypeptide antibody could specifically recognize the natural NcAP2XII-4 in the parasite, with a size of approximately 400 KDa, which was consistent with the expected size, indicating the good specificity of anti-NcAP2XII-4 antibody (Fig. 2c). The IFA test showed that NcAP2XII-4 was distributed in the nucleus, consistent with the predicted result (Fig. 2d). These results suggested that *N. caninum NcAP2XII-4* was successfully identified.

### *NcAP2XII-4* is an essential gene in* N. caninum*

**Fig. 3:**
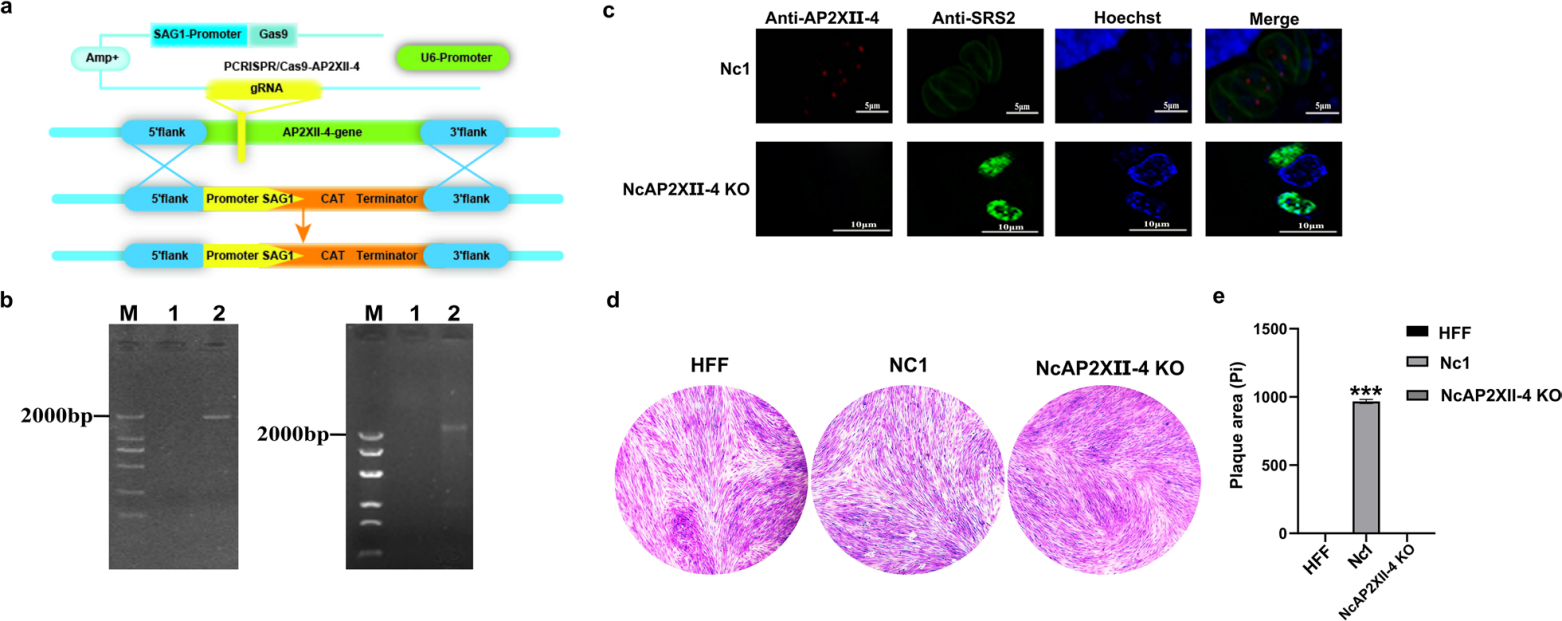
Construction and identification of NcAP2XII-4 KO parasites. **a** Schematic diagram of NcAP2XII-4 KO parasite construction. **b** Gene identification of NcAP2XII-4 KO parasites. **c** Analysis of IFA results. The vacuole formation of Nc1 parasites with the signal of AP2XII-4 (red) was normal, whereas NcAP2XII-4 KO parasites without the signal of AP2XII-4 were abnormal. **d** Plaque assay comparing growth of Nc1 and NcAP2XII-4 KO parasites. **e** The plaque areas were counted by randomly choosing 30 plaques and using the pixel point; the data were compiled from three independent experiments. “Pi” indicates pixels of each plaque

To investigate the function of *N. caninum* NcAP2XII-4, parasites deficient in NcAP2XII-4 were generated using the following method as illustrated in Fig. 3a. Using CRISPR/Cas9 technology, a pCRISPR/Cas9-AP2XII-4 plasmid with CAT was constructed and co-transfected into Nc1 with the donor DNA. Based on the preliminary evidence of specific recombination bands of 1940 bp and 2350 bp by PCR, the *NcAP2XII-4* gene was disrupted in *N. caninum* (Fig. 3b). The results of IFA indicated that Nc1 strain had an evident NcAP2XII-4 signal, while the signal was undetectable in the *NcAP2XII-4* KO strain (Fig. 3c). It is suggested that the parasite loss of *NcAP2XII-4*, which is also known as ΔNcAP2XII-4, was successful. It was discovered during the culture process of parasites. The PVs of ΔNcAP2XII-4 were formed in the host cells; however, these PVs were significantly smaller than those of Nc1, and the shapes of the parasites were abnormal (Fig. 3c). After 5 days of continuous culturing with CAT medium, it was confirmed that all parasites remained unreleased and eventually died. The plaque assay showed that Nc1 formed normal plaques, whereas ΔNcAP2XII-4 failed to produce plaques (Fig. 3d, e). Hence, we infer that *NcAP2XII-4* is an essential gene for maintaining normal life activities in *N. caninum*.

### Screening for the binding site of NcAP2XII-4

**Fig. 4:**
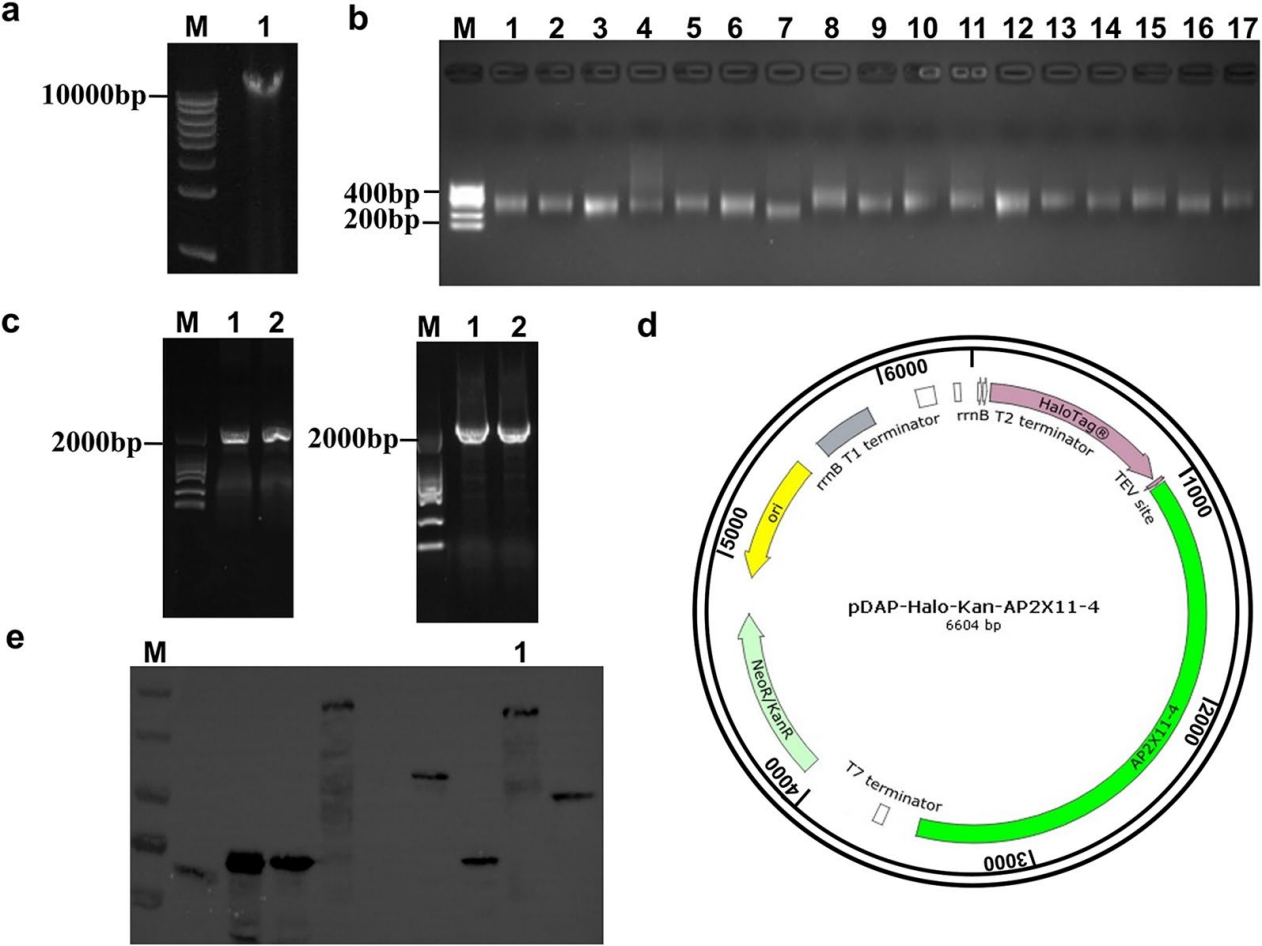
Gene library and pDAP-AP2XII–4 expression vector. **a** Genomic DNA extraction. **b** Fragmentation of genomic DNA. **c** Amplification of NcAP2XII-4 gene. **d** Construction map of the expression vector. The green region represents the APXII-4 insertion gene. **e** Western blot analysis of the protein expression vector. M: Marker; 1: target protein

Genomic DNA of *N. caninum* was successfully extracted (Fig. 4a) and fragmented (Fig. 4b). The DNA fragment containing the AP2 domain of the NcAP2XII-4 gene was amplified to a size of approximately 2400 bp (Fig. 4c). The target gene was successfully connected to the cell-free expression vector pDAP, resulting in the construction of the expression vector pDAP-AP2XII-4, which was confirmed by sequencing (Fig. 4d). Western blot showed that a 129-kDa HaloTag-labeled target protein was successfully obtained (Fig. 4e).

**Fig. 5:**
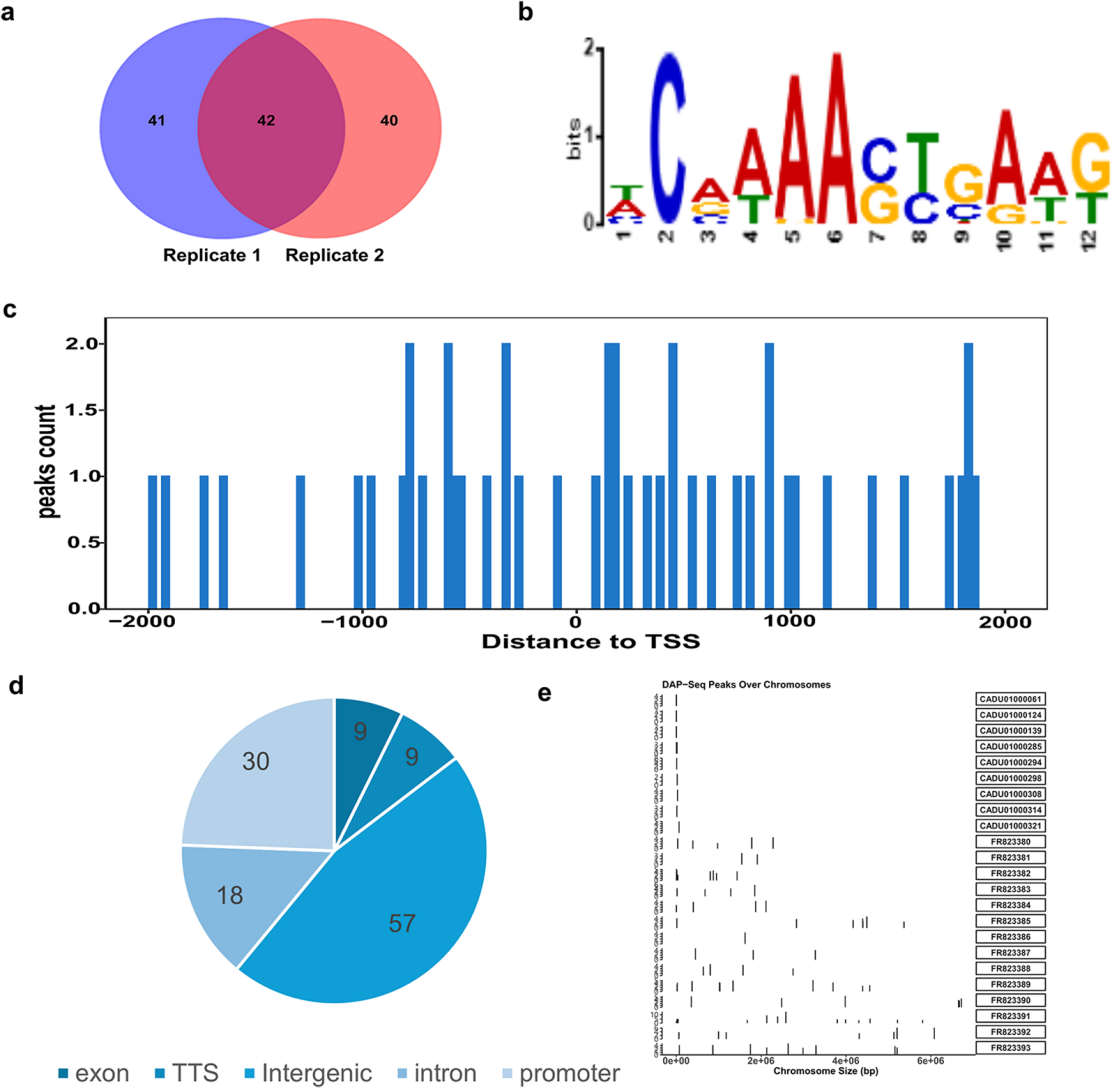
DAP-seq data analysis. **a** Venn diagram showing the number of peaks identified in Replicate 1 and Replicate 2. **b** Sequences enriched around the summits of AP2XII-4 peaks. **c** Distribution of peaks near the transcription start sites. **d** Annotation statistics of peaks in the genomic region. Pie chart showing the functional categories of target genes (123 genes in total). Hypothetical protein-encoding genes are not shown. The number of members in each group is shown in the chart. **e** Distribution of peaks on chromosomes, with the height indicating the relative mass of the peaks

The entire gene functional region is divided into promoter, exon, intron, transcription start site (TSS), transcription termination site (TTS), and intergenic, but transcription factor-binding sites are generally enriched near the TSS region. Accordingly, our attention was directed towards the peaks that had been annotated as promoters and TSS. Two independent biological replicates, replicate 1 and replicate 2, identified 83 and 82 peaks with fold enrichment > 2.0, respectively, with 42 overlapping peaks (Fig. 5a). A binding motif sequence, HCRAAASYGAWK, was identified using MME software analysis (Fig. 5b). The effective peaks were distributed both upstream and downstream of TSS (Fig. 5c) and were located on chromosomes with different numbers (Fig. 5d, e). Following the annotation of the peaks, they were mainly distributed in the intergenic regions (57%) and promoters (30%).

### Identification of the potential biological process and signaling pathways of peak-related genes

**Fig. 6:**
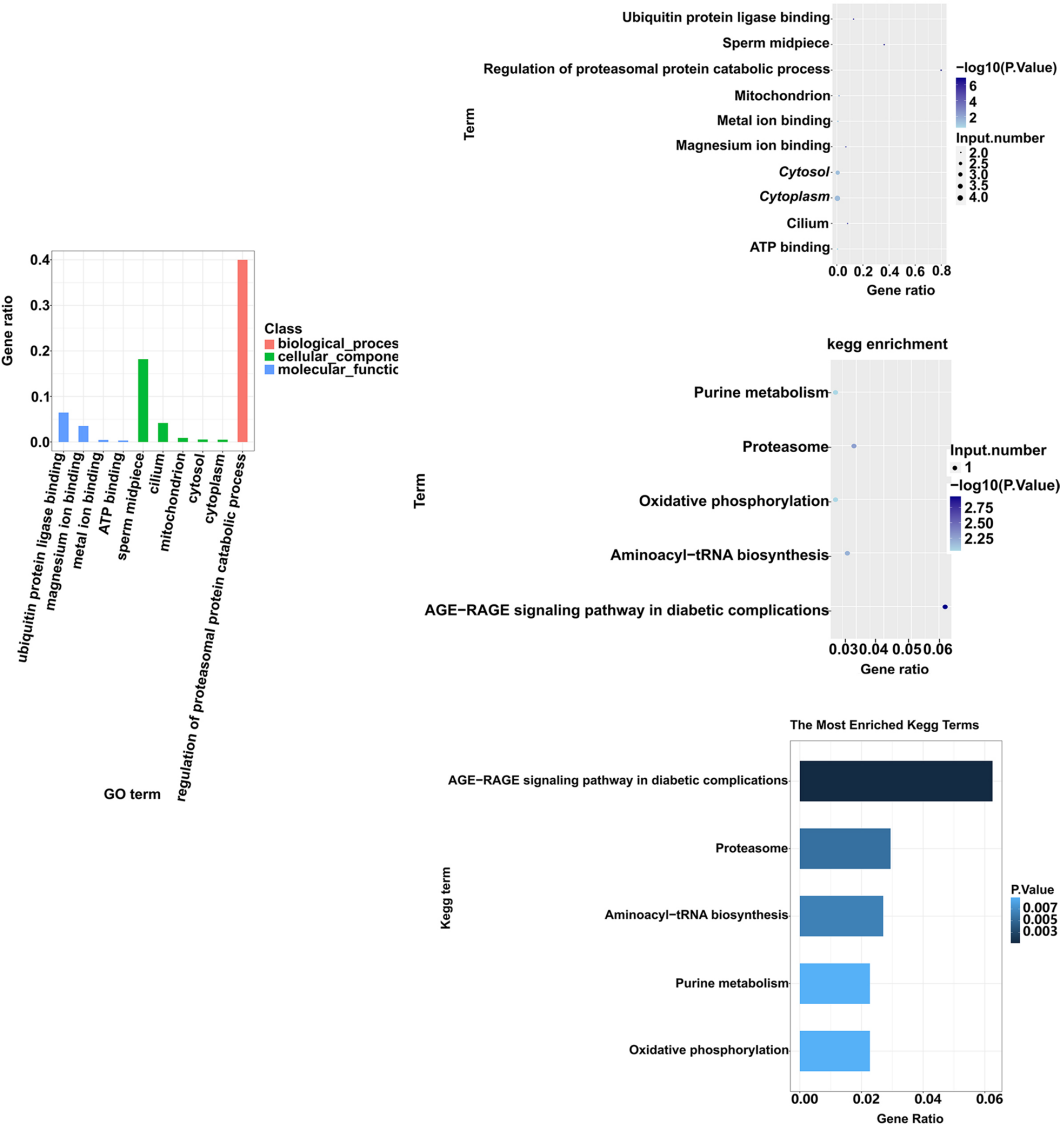
GO and KEGG analyses of peak-associated genes. **a** Bar chart analysis of the GO of peak-associated genes. **b** Bubble chart analysis of the GO enrichment of the top 20 peak-associated genes corresponding to the promoter. **c** Bar chart of the KEGG analysis of peak-associated genes. **d** Bubble chart analysis of the KEGG enrichment of the top 20 peak-associated genes corresponding to the promoter

To date, the function of NcAP2XII-4 in *N. caninum* has remained unclear. According to the GO and KEGG annotation data, all *N. caninum* genes were chosen as the background, and all genes (*P* < 0.05) related to all peaks were obtained to estimate the differential GO and KEGG pathways. GO can be divided into three parts, molecular function, biological process, and cellular components. Among these, the target genes, regulated by NcAP2XII-4, involved in biological processes were the most common, followed by those involved in cellular components and molecular function in *N. caninum* (Fig. 6a). The bubble chart analysis revealed that the genes that differ the most are those that are involved in the regulation of proteasomal protein catabolic process, sperm midpiece, and ubiquitin protein ligase binding, corresponding to the biological process, cellular component, and molecular function (Fig. 6b). Sperm midpieces may be associated with proliferation. KEGG is a database containing information on biochemical reactions, signaling pathways, metabolic pathways, and biological processes. KEGG pathway enrichment analysis was performed on the differentially expressed genes, and the results showed that the significantly differentially expressed genes were mainly enriched in the pathways of AGE-RAGE signaling pathway in diabetic complications, proteasome, aminoacyl-tRNA biosynthesis, purine metabolism, and oxidative phosphorylation (Fig. 6c, d).

### NcAP2XII-4 binding ROP5 promoter in* N. caninum*

**Fig. 7:**
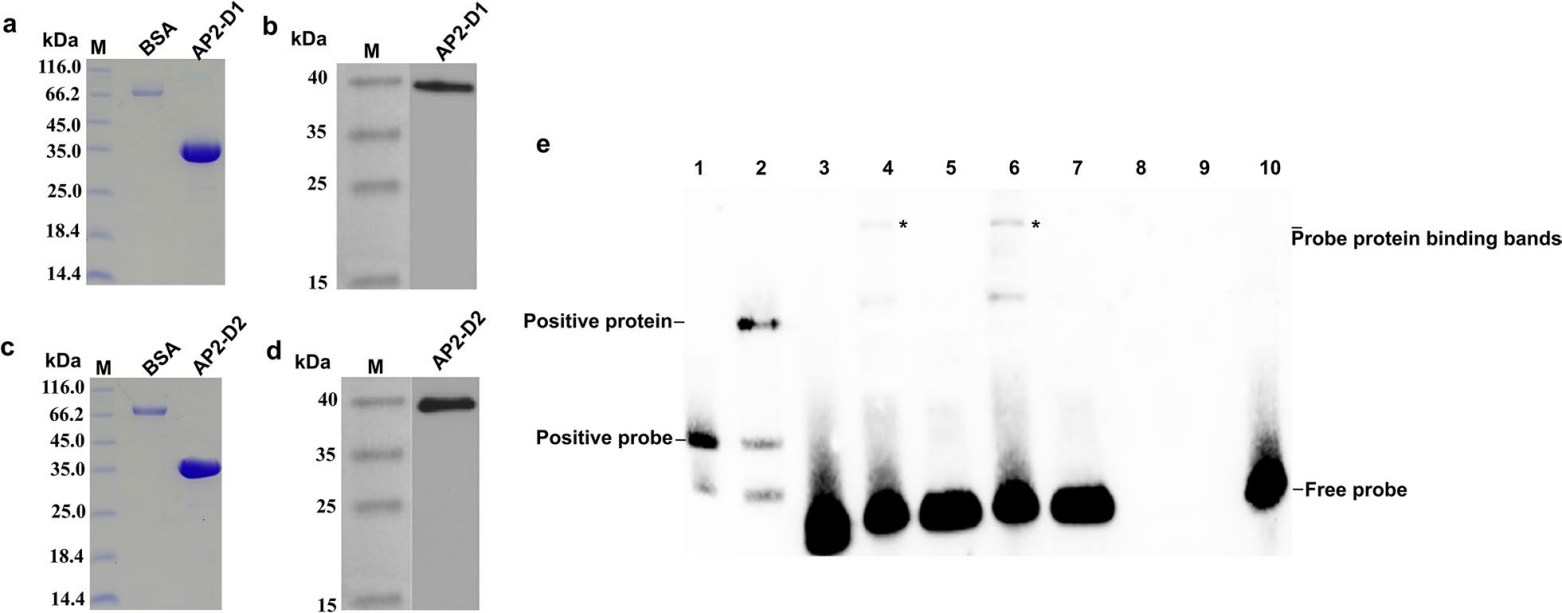
NcAP2XII-4 binding to the ROP5 promoter using EMSA assay. **a** Analysis of the AP2-D1 protein by SDS-PAGE. **b** Analysis of AP2-D1 protein by WB. **c** Analysis of the AP2-D2 protein by SDS-PAGE. **d** Analysis of AP2-D2 protein by WB. M: Marker. **e** EMSA assay. 1: Positive probe; 2: positive probe + positive protein; 3: probe; 4: probe + protein AP2XII-4-D1; 5: probe + protein AP2XII-4-D1 + probe (cold); 6: probe 1 + protein AP2XII-4-D2; 7: probe + protein AP2XII-4-D2 + probe (cold); 8: protein AP2XII-4-D1; 9: protein AP2XII-4-D2; 10: probe + HaloTag-labeled protein. *Binding protein

Previous studies showed that the expression of NcAP2XII-4 is abnormal after deletion of ROP5 [27], suggesting a potential interaction between NcAP2XII-4 and ROP5. Analysis of DAP-seq data revealed that regulation of the ROP5 promoter was found among the NcAP2XII-4 regulatory factors, indicating that NcAP2XII-4 might bind to a motif in the ROP5 promoter. To verify this, we prepared a biotin-labeled ROP5 probe and two NcAP2XII-4 proteins containing the AP2 domain, AP2XII-4-D1 (Fig. 7a), and AP2XII-4-D2 (Fig. 7c), which was successfully verified by WB, respectively (Fig. 7b, d), to perform EMSA experiments. The results showed that the target bands appeared as expected in the lanes of probe + AP2XII-4-D1 and probe + AP2XII-4-D2 but were negative in the lanes of probe (cold) + AP2XII-4-D1 and probe (cold) + AP2XII-4-D2, suggesting the specificity of the probe (Fig. 7e). In other words, the probe specifically combined AP2XII-4-D1 and AP2XII-4-D2 proteins, and there was competition between the probe and the cold probe, implying that NcAP2XII-4 could bind to the ROP5 promoter and regulate the function of ROP5 in *N. caninum*.

## Discussion

*Neospora caninum* is a protozoan that parasitizes animal cells and poses a significant threat to dairy cattle. Despite its importance, there are few reports on the transcriptional regulation of *N. caninum*. One notable report indicated that the deletion of ROP5, a key virulence factor of *N. caninum*, increases the transcription level of NcAP2XII-4 [27]. Building on this, our study successfully identified *N. caninum* NcAP2XII-4 at the gene, protein, and cell levels. We confirmed its transcriptional regulation function and described it as an essential gene of the parasite.

The AP2/ERF family of transcription factors is related to biological development and stress resistance. The *N. caninum NcAP2XII-4* gene (NCLIV_063920) was discovered in the ToxoDB database during this study. Structural predictive analysis revealed two domains similar to PF14-0633. However, the predicted protein size of approximately 410 KDa presents a challenge to expressing the full-length protein and preparing the antibody. After numerous experiments, a polypeptide antibody against NcAP2XII-4 was successfully prepared and employed to the detect NcAP2XII-4 in *N. caninum*. Historically, in most cases, the full-length protein or fragment was selected as an antigen for as long as feasible. The fusion of numerous peptides could be utilized to prepare antibodies according to the results of this study. This method provides convenience in preparing antibodies against difficult antigens, such as those with complex structures, large molecular weights, and low codon usage frequencies.

In recent years, the interaction between proteins and DNA has been widely used to study transcriptional regulation in model organisms. Previously, the combination of ChIP-seq and RNA-seq was utilized to explore the interaction. However, this technology necessitates high-quality antibodies, which presents a challenge when studying proteins with large molecular weights and obtaining high-quality antibodies. DAP-seq not only has the advantages of ChIP-seq and RNA-seq but also does not require the preparation of high-quality antibodies and even has higher sensitivity than ChIP-seq and RNA-seq. DAP-seq is particularly well suited for studying the interactions between DNA and proteins that are difficult to obtain. Currently, DAP-seq is widely used in the study of protein and DNA interactions in model plants, such as maize and arabidopsis [32]. Ning Lihua et al. [33] applied DAP-seq technology to detect 317 maize transcription factors that combined with target genes and played a role in transcription regulation. Among them, 97 target genes were detected and reported by ChIP-seq, and 220 target genes were detected by DAP-seq. The results are highly reliable. He et al. [34] investigated the transcriptional regulation function of abscisic acid and synthetic auxin 1-naphthyl acetic acid (NAA) on grape norisoprenoid. They combined weighted gene co-expression network analysis (WGCNA) and DAP-seq and identified regulatory factors of norisoprenoid production. Zhu et al. [35] identified 52 key direct targets of OsbZIP09 using RNA-seq and DAP-seq analyses, including OsLOX2 and late embryogenesis-rich (LEA) family genes involved in controlling seed germination in rice. D'Inca et al. [36] explored the transcriptional regulatory mechanism of grape VviNAC33 in terminating photosynthetic activity and organ growth using the DAP-seq method.

DAP-seq has been extensively applied to investigate transcription factors in maize, *Arabidopsis thaliana*, poplar, strain, Chinese pine, and human, with maize and rice being the most prominent examples. Furthermore, DNA-seq technology has been employed to investigate the function of bacterial proteins. Trouillon et al. [37] identified 39 genes that are mainly involved in the metabolism of *Pseudomonas aeruginosa* and are regulated by eight transcription factors using DAP-seq technology. The success rate of the DAP-seq experiment is also related to the type of transcription factor; for example, the success rate of bZIP and NAC transcription factor families is obviously higher than that of bHLH and MADs-box families [35, 38]. ChIP-seq and RNA-seq have been widely used in parasite research in the past. Although DAP-seq technology has many advantages, it has not been applied to research on the protein function of parasites. In this study, we investigated the transcriptional regulation of NcAP2XII-4 by using DAP-Seq. Notably, the motif of NcAP2XII-4 has been successfully identified with good conservation and high reliability, thus confirming the feasibility of the DAP-seq technique in the study of transcription factors of *N. caninum*. This has provided a reference method for the study of transcriptional regulation of *N. caninum* and other apicomplexans. The motif obtained from the above analysis was analyzed, and its binding protein contained rhoptry protein family factors. Previous studies have shown that the deletion of ROP5, which is a key virulence factor of *N. caninum*, causes an increase in the transcription level of NcAP2XII-4. Thus, we have prepared a protein containing the AP2 domain in *N. caninum* NcAP2XII-4 and conducted the EMSA test with a ROP5 biotin-labeled probe. The results indicate that NcAP2XII-4 is capable of binding to the promoter of ROP5 and exerting its transcriptional activity. This not only demonstrates the reliability of the results of the DAP-seq technique but also further demonstrates the feasibility of the technique in the field of parasites. This study has filled a significant gap in the research field of transcription regulation factors of the ApiAP2 family of *N. caninum*. It has laid a foundation for the research on the transcription regulation function of *N. caninum* and provided new candidate factors for the research and development of *N. caninum* vaccine.

## Conclusion

The transcription factors of *N. caninum* were identified at the gene, protein, and cell levels of NcAP2XII-4, and it was determined that the gene is an essential gene for *N. caninum* to complete its life cycle. The gene primarily functions in transcription regulation in *N. caninum* and regulates the activity of ROP5.

## Data Availability

No datasets were generated or analyzed during the current study.
